# Supplementary material for: Changes in the distribution of fitness effects and adaptive mutational spectra following a single first step towards adaptation
Source: Nat Commun. 2021 Aug 31;12:5193. doi: 10.1038/s41467-021-25440-7 (PMC8408183; doi:10.1038/s41467-021-25440-7)
Supplement: Supplementary file 1 — Supplementary Information [file 41467_2021_25440_MOESM1_ESM.pdf]

Supplementary Information

Changes in the distribution of fitness effects and adaptive mutational spectra following a single first step towards adaptation

Dimitra Aggeli, Yuping Li, Gavin Sherlock

Nature Communications

Corresponding author: Gavin Sherlock gsherloc@stanford.edu

Supplementary Note:

Various factors determine the frequency of diploids in an evolving population
Diploidy, like other types of mutation will keep feeding into the population at some underlying mutation rate. However, the constant feeding of diploids does not necessarily lead to their increased frequency for the following two reasons:

1. The frequency dynamics of diploids are determined not only by how rapidly diploids enter the population, but also by how they fluctuate once they arise. Variation in the offspring number of diploids introduces stochasticity, which can lead to diploids' extinction (drift out). If a diploid does not go extinct, its population size will eventually be large enough that it then grows exponentially and essentially deterministically: i.e. it "establishes". The number of diploids needed to avoid stochastic fluctuations is inversely proportional to the fitness value of diploids relative to the population mean fitness: *n(est) ~ 1/s*, where n(est) is the number of diploids required to reach establishment size. Thus, the higher a mutant's fitness is, the smaller the *n(est)* is.
2. Secondly and most importantly, the population mean fitness increases over time in our large and well-mixed populations; consequently, the fitness of diploids relative to the population mean fitness constantly decreases as evolution proceeds.

Below is a rough calculation related to the above two reasons:

With an effective population size of ~6E8 and an estimated diploidization rate of ~1E-5 per cell per generation in the wild-type background (similar to the estimates in [1]), we expect that ~6,000 (6E8*1E-5) diploids enter the population during each generation.

At time 0, population mean fitness is close to 0. The fitness of diploids (s) relative to the WT ancestor is ~0.04 per generation by our measurements. Given *n(est) ~ 1/s,* diploids will get established in the population as long as >25 diploids (=1/0.04) enter the population, a number much lower than the 6,000. Thus, at time 0+1, diploids establish and increase their frequency at a rate of *e^(s*t)*.

Over time, other beneficial mutations will also establish and rise in frequency, and together with the diploids will increase the population mean fitness. Due to this increased population mean fitness, the relative fitness of diploids decreases and newly arising diploids are less and less likely to become established. Once the population mean fitness reaches ~0.04 (relative to WT ancestor), the effective relative fitness of diploids becomes 0 (or neutral), meaning that newly arising diploids cease to establish in the population, despite their high mutation rate (the constant feeding). Consistent with the above explanation, we observed that in our wild-type evolving population, the population mean fitness approaches 0.04 between 90-100 generations, which coincided with a decline in the overall frequency of diploids, indicating that new diploids (with no additional mutations) no longer establish (Supplementary Figure 4B, and Figure S7 in [2]); note, diploids do subsequently further increase in frequency after that dip, but due to second beneficial mutations occurring on a diploid background. Another point of note, is that the above overestimates the likelihood of establishment and observation of diploids – newly arising beneficial mutations also have an establishment time (the time they take to reach establishment size from when they arose), which further limits their likelihood of observation, because by the time they reach establishment, the population mean fitness may have already passed them by (see Figure 3 in [3]).

Supplementary Tables

Evolved strain*	Derived strain**	Evolved mutation	Effect	Pathway	Fitness evolved* (ref. 2)	Fitness evolved remeasurements*	Fitness evolved*	Fitness derived**	Location	Clone ID (ref. 2)	Barcode ID in evolution (ref. 3)
GSY5481	GSY6701	$cyr1^{S917Y}$	GOF	Ras PKA	9.82	7.96	3.63	3.47	AdapsPlate3-WellF10	131025-1-A6	43,692
GSY5128	GSY6702	$gpb2^{Y282*}$	LOF	Ras PKA	10.12	8.79	5.88	5.96	AdapsPlate1-WellC12	131007-1-D2	7,774
GSY5153	GSY6703	$tor1^{F1712L}$	LOF	TOR Sch9	6.21	4.68	4.64	4.93	AdapsPlate1-WellB09	130719-1-A5	21,543

Supplementary Table 1. Fitness and identifiers of evolved and derived strains

*correspond to the evolved parent in Supp Figure Founder fitness
**correspond to the 2nd ‘X’ with evolved variant in Supp Figure Founder fitness
generations 8, 24, 40 were used for fitness estimation of the *cyr1* mutation
generations 0, 8, 24, 40 were used for fitness estimations of the *gpb2* and *tor1* mutations

Generation	WT.evo1	WT.evo2	*cyr1*.evo1	*cyr1*.evo2	*gpb2*.evo1	*gpb2*.evo2	*tor1*.evo1	*tor1*.evo2
0	0.010	0.010	0.010	0.023	0.000	0.000	0.036	0.018
8	0.000	0.021		0.034	0.012	0.053	0.027	
16	0.010	0.011	0.020	0.020	0.000	0.051	0.019	0.070
24	0.021	0.042	0.017	0.032	0.000	0.052	0.041	0.049
32	0.042	0.031		0.090	0.024	0.043	0.043	0.063
40	0.052	0.021		0.045	0.013	0.055	0.096	0.118
48	0.073	0.031	0.013	0.063	0.038	0.037	0.106	0.092
56	0.083	0.125	0.051	0.085	0.026	0.076	0.127	0.163
64	0.198	0.104		0.083	0.031	0.071	0.156	0.151
72	0.083	0.177		0.132	0.038	0.113	0.268	0.188
80	0.219	0.240	0.076	0.097	0.044	0.069	0.267	0.329
88	0.375	0.260	0.121	0.191	0.125	0.073	0.431	0.462
96	0.531	0.396		0.177	0.145	0.157	0.479	
104	0.469	0.427	0.193	0.268	0.208	0.097	0.605	
112	0.542	0.458	0.263	0.264	0.129	0.266	0.678	
120	0.521	0.313	0.325	0.395	0.221	0.217	0.844	
128	0.417	0.365	0.385	0.401	0.187	0.333	0.935	
136	0.323	0.239		0.448	0.131	0.295	0.956	0.857
144	0.389	0.167	0.451	0.527	0.072	0.478	0.961	0.888
152	0.476	0.135	0.421	0.485	0.058	0.459	0.948	0.850
160			0.444	0.578	0.061	0.512	0.958	0.883
168	0.438	0.177						

Supplementary Table 2. Benomyl sensitivity fractions for individual clones per timepoint and evolution. For comparison, average diploid fractions at generation 88 are 0.318, 0.156, 0.099 and 0.466 for wild-type, *cyr1*, *gpb2* and *tor1* evolutions, respectively.

Name	Sequence	Usage
TOR1f	CGCCAGAGATTTGCTTGTGACTG	*tor1* locus amplification
TOR1r	CAACTGTGGTAAGACTTCAAGCCAG	*tor1* locus amplification
CYR1f	GAAAGTCAGACCCACTTCATATTATGGGT	*cyr1* locus amplification
CYR1r	ATACTCTAGTTTGTTTCTCGCCACTGA	*cyr1* locus amplification
GPB2f2	TCTACCATCTATACCGTCGGCGT	*gpb2* locus amplification
GPB2r2	TGCTGCTACCTTGTTTCATGGAAG	*gpb2* locus amplification
BC_F–DY	CAACCTGAAGTCTAGGTCCCTATT	amplification of the low complexity barcode
BC_R1–DY	GTTCTTTGCTTTTTTTCCCCAACGACGTCGAACACATTAGTCCTACGCACTTAACTTCGCATCTG	amplification of the low complexity barcode
P104	ACACTCTTTCCCTACACGACGCTCTTCCGATCTNNNNNNNNCGATGTTTAATATGGACTAAAGGAGGCTTTT	forward primer for 1st step BC library preparation
P105	ACACTCTTTCCCTACACGACGCTCTTCCGATCTNNNNNNNNACAGTGTTAATATGGACTAAAGGAGGCTTTT	forward primer for 1st step BC library preparation
P111	ACACTCTTTCCCTACACGACGCTCTTCCGATCTNNNNNNNNTGACCATTAATATGGACTAAAGGAGGCTTTT	forward primer for 1st step BC library preparation
P112	ACACTCTTTCCCTACACGACGCTCTTCCGATCTNNNNNNNNGCCAATTTAATATGGACTAAAGGAGGCTTTT	forward primer for 1st step BC library preparation
P122	ACACTCTTTCCCTACACGACGCTCTTCCGATCTNNNNNNNNATCACGTTAATATGGACTAAAGGAGGCTTTT	forward primer for 1st step BC library preparation
P123	ACACTCTTTCCCTACACGACGCTCTTCCGATCTNNNNNNNNCAGATCTTAATATGGACTAAAGGAGGCTTTT	forward primer for 1st step BC library preparation
P124	ACACTCTTTCCCTACACGACGCTCTTCCGATCTNNNNNNNNGGCTACTTAATATGGACTAAAGGAGGCTTTT	forward primer for 1st step BC library preparation
P125	ACACTCTTTCCCTACACGACGCTCTTCCGATCTNNNNNNNNTAGCTTTTAATATGGACTAAAGGAGGCTTTT	forward primer for 1st step BC library preparation
P130	ACACTCTTTCCCTACACGACGCTCTTCCGATCTNNNNNNNNTTAGGCTTAATATGGACTAAAGGAGGCTTTT	forward primer for 1st step BC library preparation
P131	ACACTCTTTCCCTACACGACGCTCTTCCGATCTNNNNNNNNACTTGATTAATATGGACTAAAGGAGGCTTTT	forward primer for 1st step BC library preparation
P132	ACACTCTTTCCCTACACGACGCTCTTCCGATCTNNNNNNNNGATCAGTTAATATGGACTAAAGGAGGCTTTT	forward primer for 1st step BC library preparation
P133	ACACTCTTTCCCTACACGACGCTCTTCCGATCTNNNNNNNNCTTGTATTAATATGGACTAAAGGAGGCTTTT	forward primer for 1st step BC library preparation
P101	CTCGGCATTCCTGCTGAACCGCTCTTCCGATCTNNNNNNNNTATATACGCTCGAATTCAAGCTTAGATCTGATA	reverse primer for 1st step BC library preparation
P108	CTCGGCATTCCTGCTGAACCGCTCTTCCGATCTNNNNNNNNCGCTCTATCTCGAATTCAAGCTTAGATCTGATA	reverse primer for 1st step BC library preparation
P109	CTCGGCATTCCTGCTGAACCGCTCTTCCGATCTNNNNNNNNGAGACGTCTTCGAATTCAAGCTTAGATCTGATA	reverse primer for 1st step BC library preparation
P110	CTCGGCATTCCTGCTGAACCGCTCTTCCGATCTNNNNNNNNATACTGCGTTCGAATTCAAGCTTAGATCTGATA	reverse primer for 1st step BC library preparation
P126	CTCGGCATTCCTGCTGAACCGCTCTTCCGATCTNNNNNNNNACTAGCAGATCGAATTCAAGCTTAGATCTGATA	reverse primer for 1st step BC library preparation
P127	CTCGGCATTCCTGCTGAACCGCTCTTCCGATCTNNNNNNNNTGAGCTAGCTCGAATTCAAGCTTAGATCTGATA	reverse primer for 1st step BC library preparation
P128	CTCGGCATTCCTGCTGAACCGCTCTTCCGATCTNNNNNNNNCTGCTACTCTCGAATTCAAGCTTAGATCTGATA	reverse primer for 1st step BC library preparation
P129	CTCGGCATTCCTGCTGAACCGCTCTTCCGATCTNNNNNNNNGCGTACGCATCGAATTCAAGCTTAGATCTGATA	reverse primer for 1st step BC library preparation
PE1	AATGATACGGCGACCACCGAGATCTACACTCTTTCCCTACACGACGCTCTTCCGATCT	forward primer for 2nd step BC library preparation
PE2	CAAGCAGAAGACGGCATACGAGATCGGTCTCGGCATTCCTGCTGAACCGCTCTTCCGATCT	reverse primer for 2nd step BC library preparation

Supplementary Table 3. Oligos used in the current study

Supplementary Figures

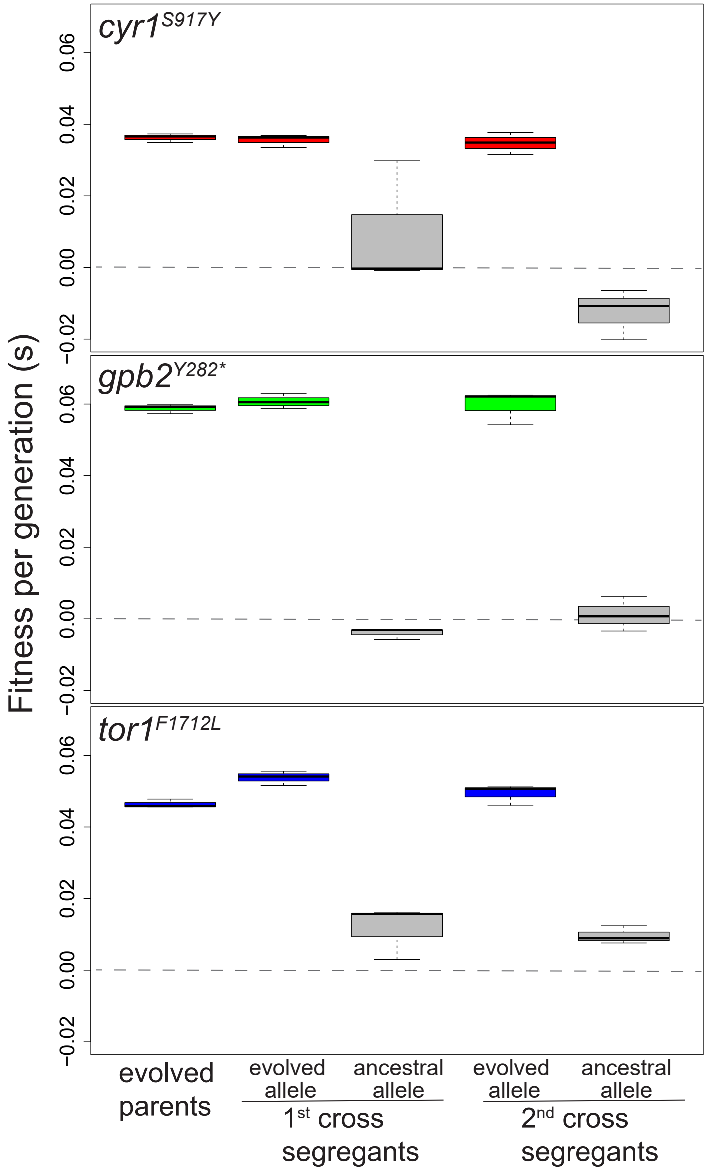

Supplementary Figure 1. The fitness advantage is a monogenic trait and co-segregates with the evolved variants. Fitness advantage was measured against a fluorescent version of the initial ancestor, for each parental evolved clone and for 4 segregants derived from 2 consecutive back-crosses of the evolved clone with their ancestor. From each cross, one segregant with the evolved variant and one segregant with the ancestral variant were assayed. Fitness was measured in triplicates. Generations 8, 24 and 40 were used for fitness estimation of the *cyr1* mutant and derivatives. Generations 0, 8, 24 and 40 were used for fitness estimations of the *gpb2* and *tor1* mutants and derivatives. The lower and upper hinges of each box correspond to the first and third quartiles (the 25th and 75th percentiles). The bold line inside the rectangular defines the median. The whiskers extend from the hinge to a value no further than 1.5 * IQR from the hinge (where IQR is the inter-quartile range). 3 replicate assays per boxplot were used. Fitness values are provided in the Source Data folder.

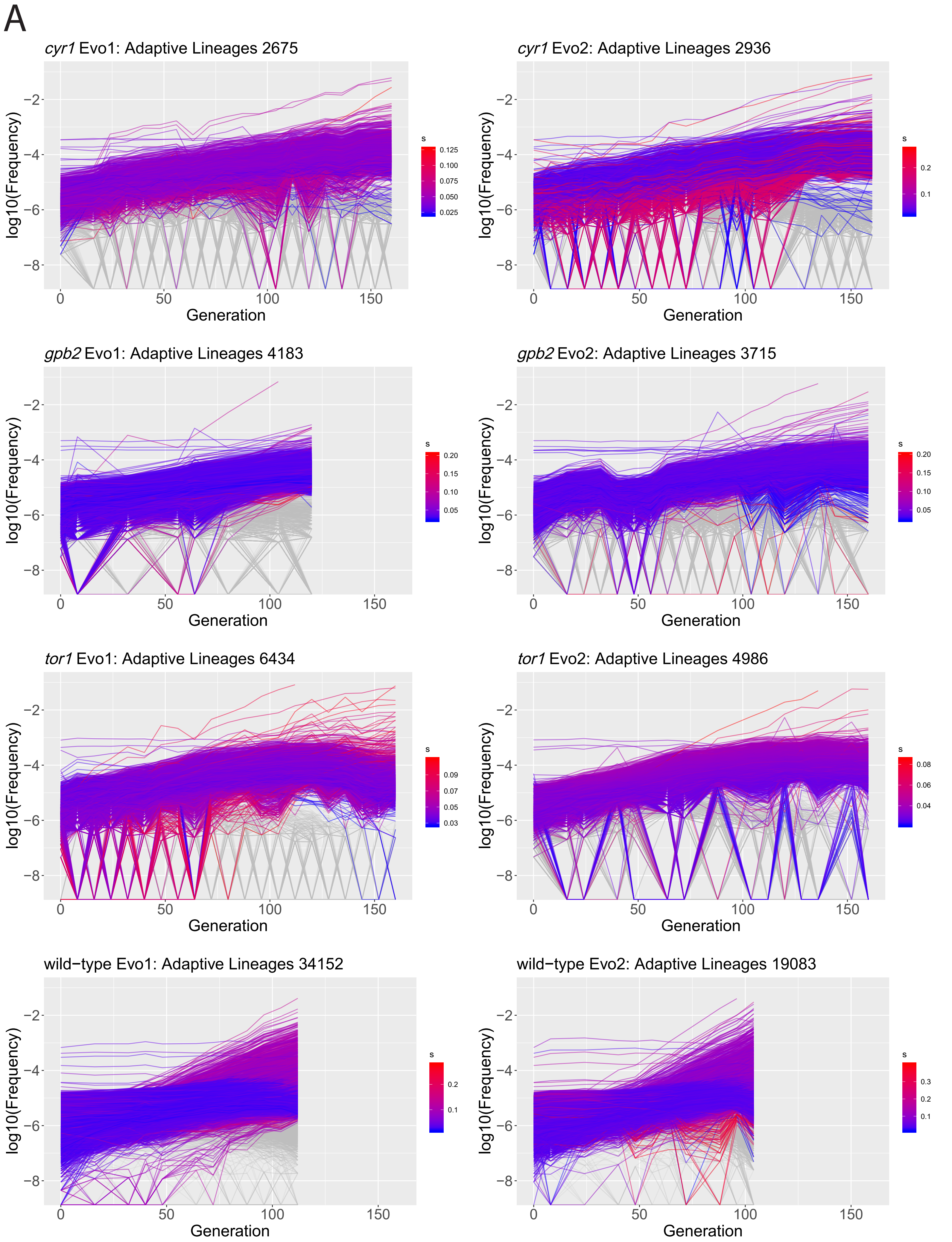
A
cyr1 Evo1: Adaptive Lineages 2675
cyr1 Evo2: Adaptive Lineages 2936
gpb2 Evo1: Adaptive Lineages 4183
gpb2 Evo2: Adaptive Lineages 3715
tor1 Evo1: Adaptive Lineages 6434
tor1 Evo2: Adaptive Lineages 4986
wild−type Evo1: Adaptive Lineages 34152
wild−type Evo2: Adaptive Lineages 19083
log10(Frequency)
Generation
s

B

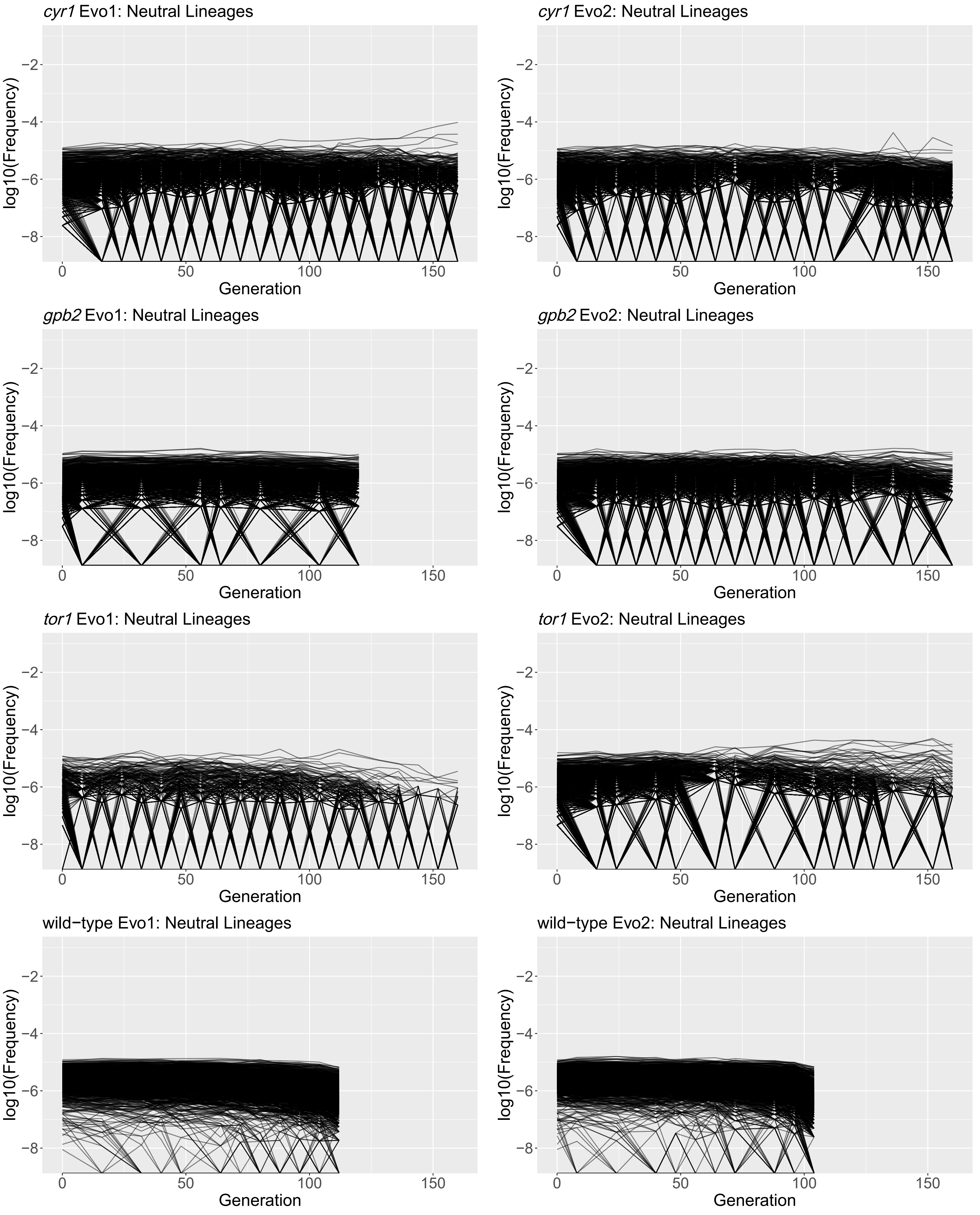

cyr1 Evo1: Neutral Lineages
cyr1 Evo2: Neutral Lineages
gpb2 Evo1: Neutral Lineages
gpb2 Evo2: Neutral Lineages
tor1 Evo1: Neutral Lineages
tor1 Evo2: Neutral Lineages
wild−type Evo1: Neutral Lineages
wild−type Evo2: Neutral Lineages
log10(Frequency)
Generation
−2
−4
−6
−8
0
50
100
150

Supplementary Figure 2. Lineage trajectories over the course of the evolutions. Lineage frequencies were estimated from time course barcode sequencing data. Data from the wild-type evolutions were subsampled from the counts in supplemental datasets 1 and 2 from [4]. **A.** Highlighted trajectories represent lineages with a fitness advantage and are colored by fitness. Grey trajectories represent neutral lineages, which are also shown in **B.** for clarity. In all cases 1,000 neutral trajectories are shown. Evo1 and Evo2 refer to replicate evolution experiments 1 and 2. Barcode counts per evolution and fitness information for the adapted ancestors are provided in the Source Data folder.

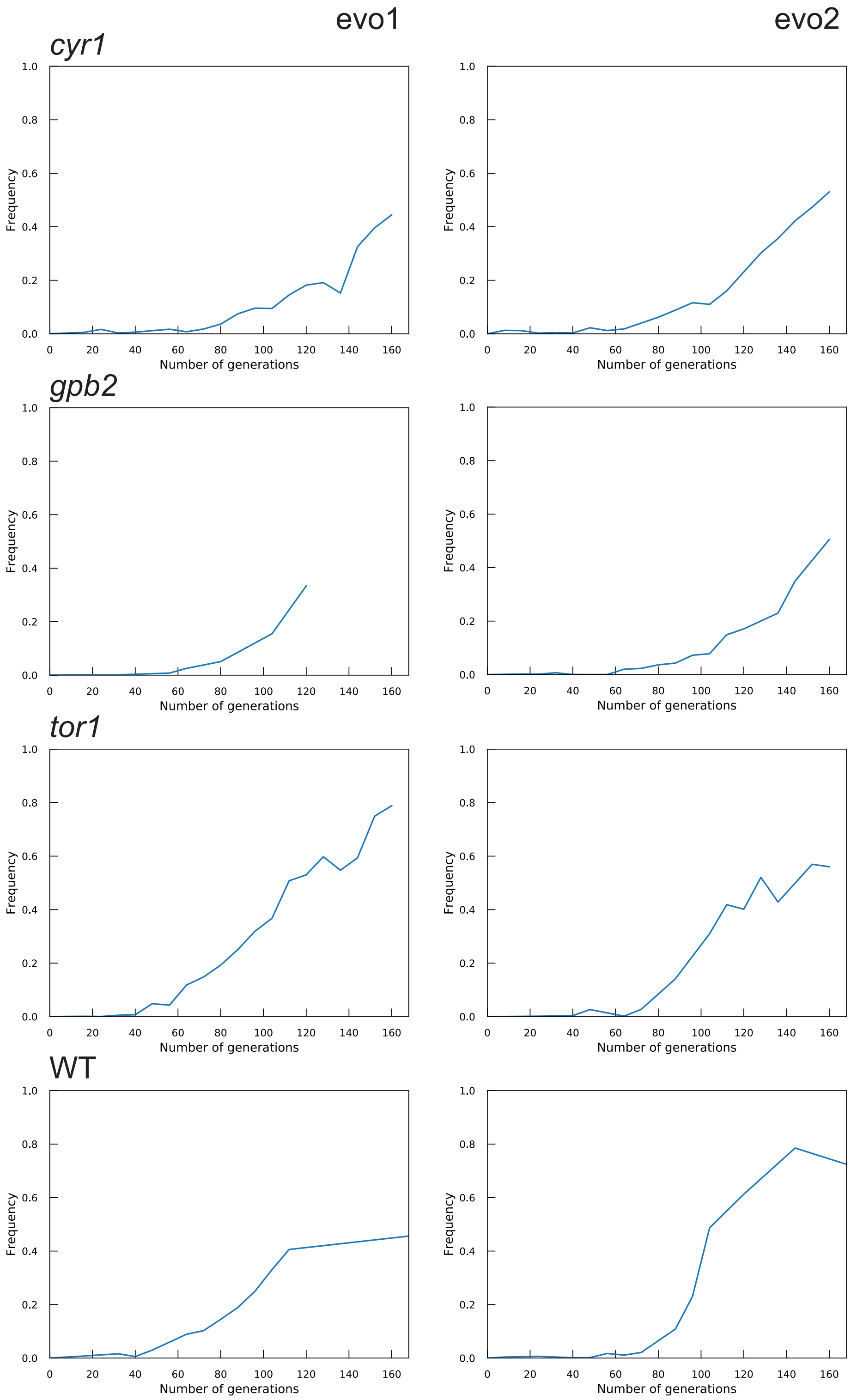

Supplementary Figure 3. Frequency of adaptive individuals over the course of the evolutions. The frequency was calculated using the barcode sequencing data. Wild-type is annotated as WT.

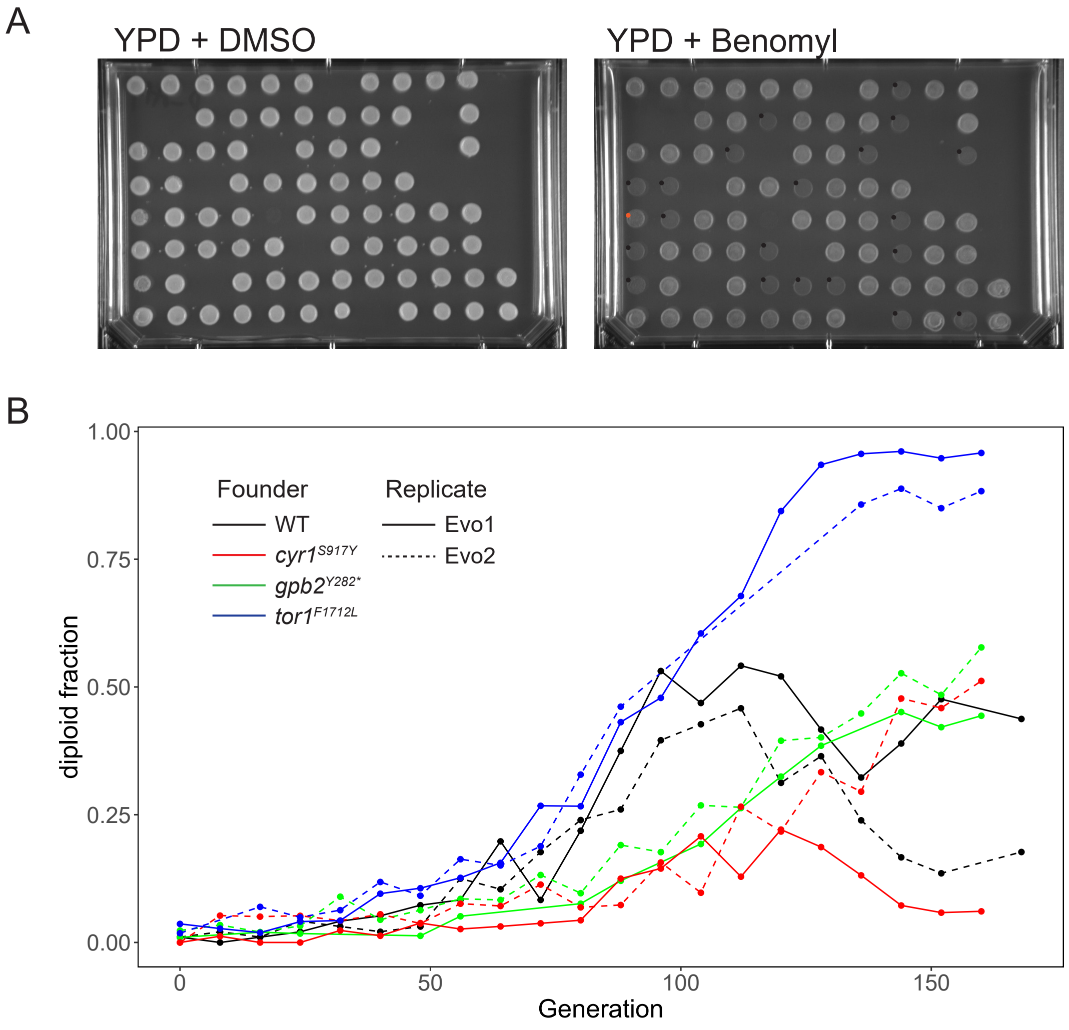

Supplementary Figure 4. Diploid trajectories over the course of the evolutions. The diploid fraction was estimated via a benomyl-based ploidy assay. **A).** Representative pair of plates that were used to score diploidy prevalence at each evolution and timepoint. Empty wells are because of instrument cell sorting. Clones annotated with a black dot were scored as diploids. The clone annotated with an orange dot was not included in the calculation. The rest were scored as haploids. **B).** Diploid fractions over time for each of the evolutions. Data of the wild-type (annotated WT) evolution are the same as in [2], figure S8. Raw data are provided in Supplementary Data 1 Table 4.

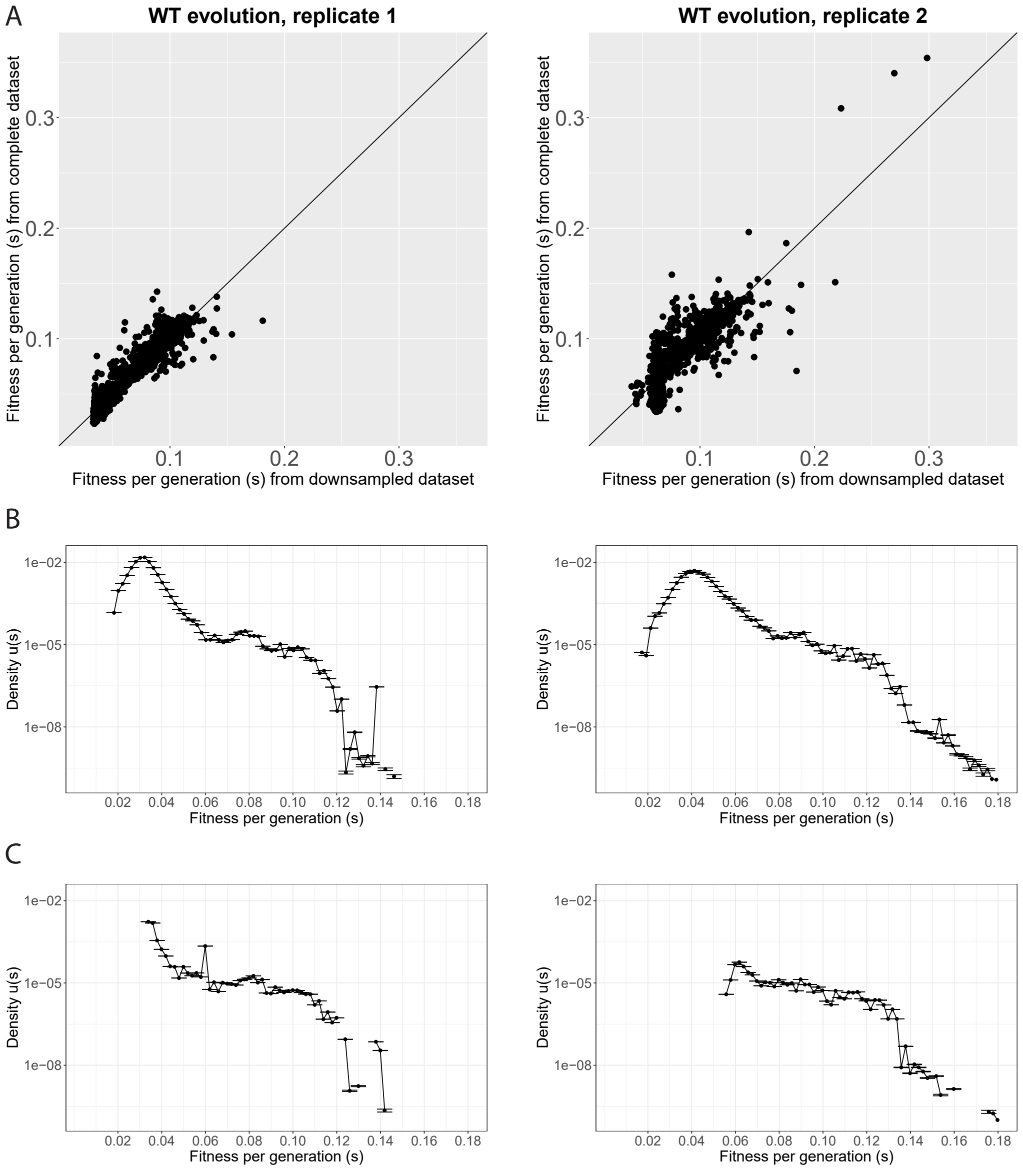

Supplementary Figure 5. Downsampling of sequencing data does not affect the fitness calculation but affects the detection limit. Barcode sequencing data from the 1st-step evolutions (WT; wild-type) were downsampled to depths comparable to the data from the 2nd-step evolutions. **A.** Fitness effects were estimated using the complete and the downsampled datasets for both replicates[4]. **B-C.** Mutation rates per fitness bin as calculated using the complete (B) and the downsampled (C) datasets. The Y-axis error is defined in equation (2) and the error bars define the mutation rate +/- the error. Fitness values and mutation rates are provided in the Source Data folder for Supplementary Fig. 5 and Fig. 2, respectively.

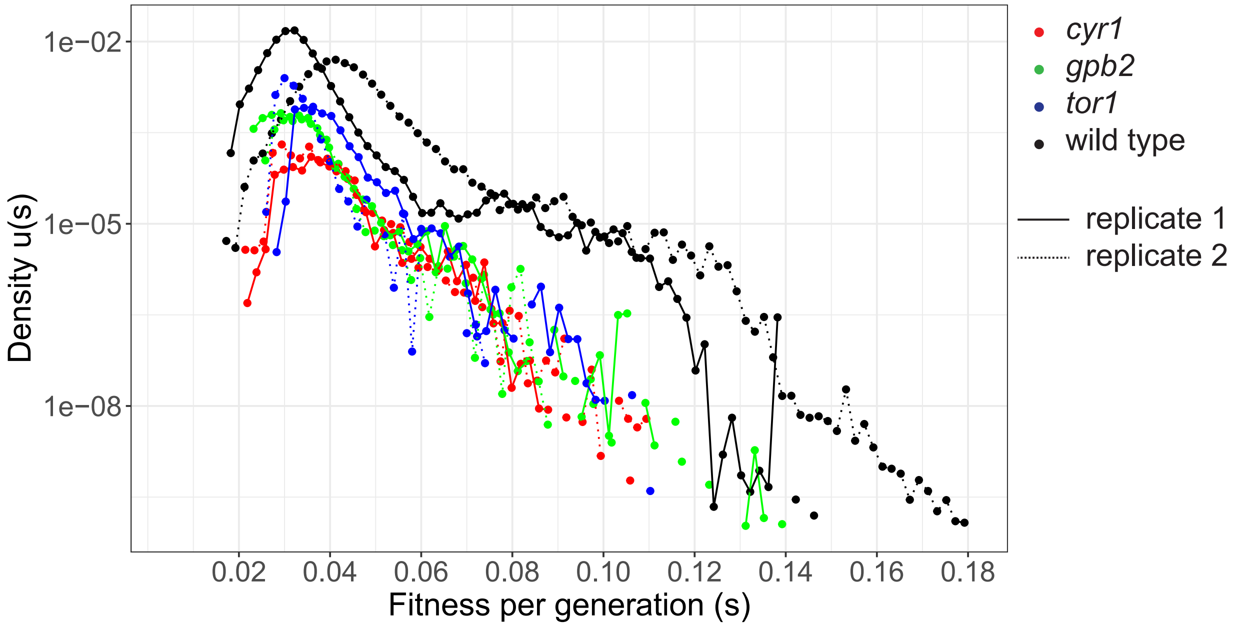

Supplementary Figure 6. Mutation rates and fitness effects are smaller during the 2nd-step evolutions, as compared to the 1st-step. Direct comparison of all genotypes. Mutation rates per fitness bin were calculated for all 2nd-step evolutions and compared to the 1st-step (wild type). The same data plotted in Fig. 2 are shown here in a single plot for comparison of all genotypes. Error bars are omitted. Mutation rates per fitness interval are provided in the Source Data folder.

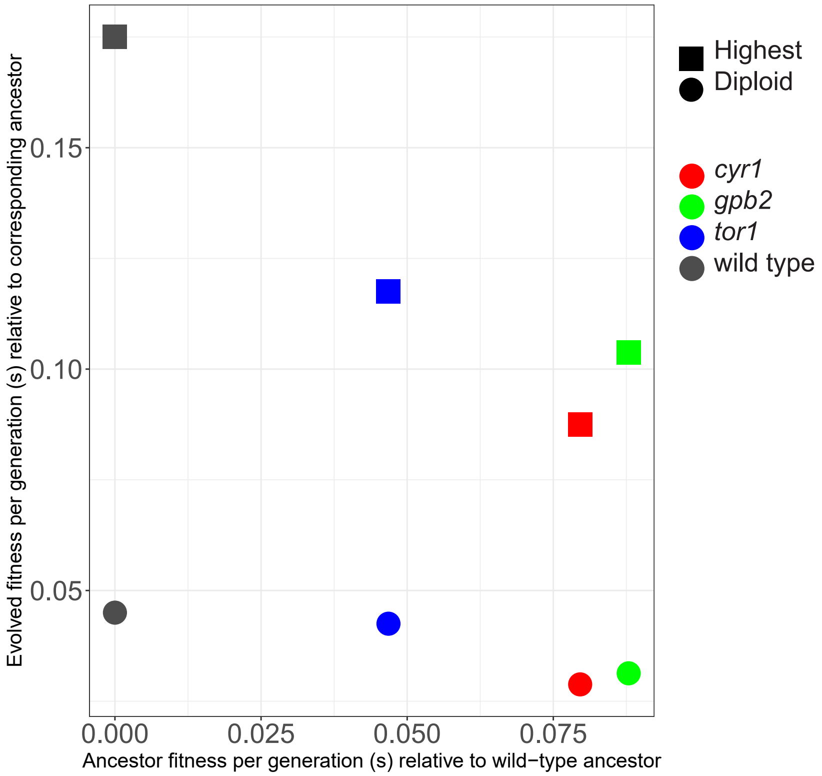

Supplementary Figure 7. Highest and diploidization fitness gains anticorrelate with ancestor fitness. Fitness effects are expressed per generation. Pearson correlations (two-sided) equal -0.95 (p-value = 0.049) and -0.91 (p-value = 0.091), for highest and diploidization fitness effects, respectively, and Spearman correlations (two-sided) equal -0.8 (p-value = 0.333) for both.

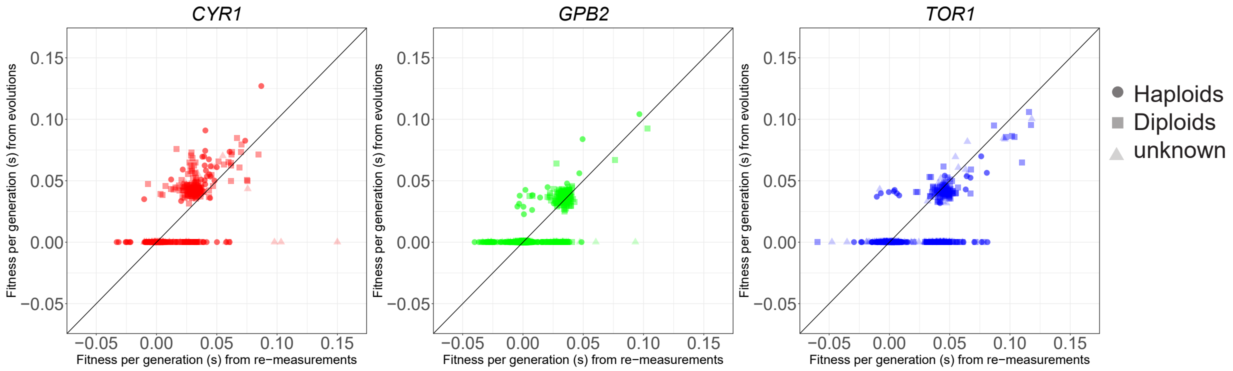

Supplementary Figure 8. Fitness estimations from remeasurements of individual clones match the fitness values inferred from lineage tracking data of the evolutions. Fitness values were matched based on barcode identity and ploidy characterization was based on a benomyl assay for individual clones. Pearson correlations (two-sided) equal 0.558, 0.597 and 0.747 (p-value < 2.2e-16 in all cases), for *cyr1*, *gpb2* and *tor1* clones, respectively. Clones with an apparent 0 value on the y-axis did not have fitness value inferred from the evolution data and were not included in the correlation calculation. Fitness values are provided in the Source Data folder and are the same as in Fig. 3 and Supplementary Fig. 2.

References

1. Harari, Y., Ram, Y., Rappoport, N., Hadany, L. & Kupiec, M. Spontaneous Changes in Ploidy Are Common in Yeast. *Current Biology* **28**, 825-835.e4 (2018).
2. Venkataram, S. *et al.* Development of a Comprehensive Genotype-to-Fitness Map of Adaptation-Driving Mutations in Yeast. *Cell* **166**, 1585-1596.e22 (2016).
3. Levy, S. F. *et al.* Quantitative evolutionary dynamics using high-resolution lineage tracking. *Nature* **519**, 181–186 (2015).
4. Blundell, J. R. *et al.* The dynamics of adaptive genetic diversity during the early stages of clonal evolution. *Nat Ecol Evol* **3**, 293–301 (2019).
