# Supplementary material for: Changes in the distribution of fitness effects and adaptive mutational spectra following a single first step towards adaptation
Source: Nat Commun. 2021 Aug 31;12:5193. doi: 10.1038/s41467-021-25440-7 (PMC8408183; doi:10.1038/s41467-021-25440-7)
Supplement: Supplementary file 3 — Descriptions of Additional Supplementary Files [file 41467_2021_25440_MOESM3_ESM.pdf]

Descriptions of Additional Supplementary Files

Supplementary data 1
Description: Read counts and total barcodes for timepoints included in lineage tracking per evolution.
