# Supplementary material for: Changes in the distribution of fitness effects and adaptive mutational spectra following a single first step towards adaptation
Source: Nat Commun. 2021 Aug 31;12:5193. doi: 10.1038/s41467-021-25440-7 (PMC8408183; doi:10.1038/s41467-021-25440-7)
Supplement: Supplementary file 5 — Reporting Summary [file 41467_2021_25440_MOESM5_ESM.pdf]

nature research

Corresponding author(s): Gavin Sherlock

Last updated by author(s): Dec 14, 2020

Reporting Summary

Nature Research wishes to improve the reproducibility of the work that we publish. This form provides structure for consistency and transparency in reporting. For further information on Nature Research policies, see our Editorial Policies and the Editorial Policy Checklist.

Statistics

For all statistical analyses, confirm that the following items are present in the figure legend, table legend, main text, or Methods section.

n/a	Confirmed	
☐	☒	The exact sample size (n) for each experimental group/condition, given as a discrete number and unit of measurement
☐	☒	A statement on whether measurements were taken from distinct samples or whether the same sample was measured repeatedly
☐	☒	The statistical test(s) used AND whether they are one- or two-sided<br>*Only common tests should be described solely by name; describe more complex techniques in the Methods section.*
☒	☐	A description of all covariates tested
☒	☐	A description of any assumptions or corrections, such as tests of normality and adjustment for multiple comparisons
☐	☒	A full description of the statistical parameters including central tendency (e.g. means) or other basic estimates (e.g. regression coefficient) AND variation (e.g. standard deviation) or associated estimates of uncertainty (e.g. confidence intervals)
☐	☒	For null hypothesis testing, the test statistic (e.g. F, t, r) with confidence intervals, effect sizes, degrees of freedom and P value noted<br>*Give P values as exact values whenever suitable.*
☒	☐	For Bayesian analysis, information on the choice of priors and Markov chain Monte Carlo settings
☒	☐	For hierarchical and complex designs, identification of the appropriate level for tests and full reporting of outcomes
☐	☒	Estimates of effect sizes (e.g. Cohen's d, Pearson's r), indicating how they were calculated

Our web collection on statistics for biologists contains articles on many of the points above.

Software and code

Policy information about availability of computer code

Data collection	*Provide a description of all commercial, open source and custom code used to collect the data in this study, specifying the version used OR state that no software was used.*
Data analysis	https://github.com/Sherlock-Lab/Barcode_seq/blob/master/bartender_BC1_BC2.py custom code used to assemble lineage trajectories from timecourse barcode sequencing data from the evolutions. Modifications of the code are described in the materials and methods and the updated code is provided with the raw lineage sequencing data submitted on SRA. It uses the software bartender, v1.1<br>The same data were used to estimate lineage fitness and establishment time with code that was used previously in Levy et al., Nature 2015 (ref. 42).<br>Fitness estimation from barcode-based bulk competition assays was performed with software in https://github. com/barcoding-bfa/fitness-assay-python, previously described in Venkataram et al., Cell 2016 (ref. 4).<br>Whole genome sequencing data were processed with cutadapt version 1.16 (fastq trimming) and bwa (alignment to reference). Variant calling and annotation was performed with https://github.com/liyuping927/DNAscope-variants-calling, which also makes use of snpEff and SNPSift.

For manuscripts utilizing custom algorithms or software that are central to the research but not yet described in published literature, software must be made available to editors and reviewers. We strongly encourage code deposition in a community repository (e.g. GitHub). See the Nature Research guidelines for submitting code & software for further information.

Data

Policy information about availability of data

All manuscripts must include a data availability statement. This statement should provide the following information, where applicable:

- Accession codes, unique identifiers, or web links for publicly available datasets
- A list of figures that have associated raw data
- A description of any restrictions on data availability

Sequencing data can be found in http://www.ncbi.nlm.nih.gov/bioproject/641174, submissionID: SUB7646176, BioProject ID PRJNA641174 and under biosamples SAMN15345397, SAMN15345398, SAMN15345399, SAMN15345400.
Raw data are provided for figures 2/ S2 (timecourse raw barcode counts) and figure 3 (fitness estimates with respect to wild-type ancestor and respective immediate ancestors)

Field-specific reporting

Please select the one below that is the best fit for your research. If you are not sure, read the appropriate sections before making your selection.

☐ Life sciences ☐ Behavioural & social sciences ☒ Ecological, evolutionary & environmental sciences

For a reference copy of the document with all sections, see nature.com/documents/nr-reporting-summary-flat.pdf

Ecological, evolutionary & environmental sciences study design

All studies must disclose on these points even when the disclosure is negative.

Study description	3 closely related yeast genotypes were passaged 20 times (160 generations) in a defined environment. After passaging clones were isolated, and their fitness and genotypes were determined
Research sample	For all experiments Saccharomyces cerevisiae derivative of the laboratory strain S288C was used that had been manipulated to be amenable to barcoding (Levy et al Nature 2015 - ref 42)
Sampling strategy	Evolution timepoints from which clones were isolated were determined based on the fraction of diploids, as well as the overall diploid trajectories. The number of clones sorted was initially set randomly to ~900 per evolution analyzed. Fitness and genotype determinations suggested that the throughput used was sufficient to yield informative results.
Data collection	Clone sorting was performed by technician at the FACS facility at Stanford. Next generation sequencing was done on Next Seq 500/550 platforms. Evolution experiments were performed by DA. Fitness remeasurements were performed by YL
Timing and spatial scale	Evolution experiments were started at November 10 2016 and ended December 21 2016. Transfers were performed every other day, cells were saved from all evolutions and timepoints, and stored at -80C (to isolate clones for ploidy determination and adaptation characterization and to restart the evolutions in case of contamination), and at -20C for gDNA preparation for timecourse barcode sequencing.
Data exclusions	Timepoints that were represented by low coverage barcode sequencing were omitted from the evolutions (shown in table S3) as well as from the fitness remeasurement assays via thresholds set by the software that was used to perform the analysis.
Reproducibility	Fitness assays were performed in triplicates.
Randomization	N/A, not random samples were collected
Blinding	N/A, not random samples were collected

Did the study involve field work? ☐ Yes ☒ No

Reporting for specific materials, systems and methods

We require information from authors about some types of materials, experimental systems and methods used in many studies. Here, indicate whether each material, system or method listed is relevant to your study. If you are not sure if a list item applies to your research, read the appropriate section before selecting a response.

Materials & experimental systems

n/a	Involved in the study
☒	☐ Antibodies
☐	☒ Eukaryotic cell lines
☒	☐ Palaeontology and archaeology
☒	☐ Animals and other organisms
☒	☐ Human research participants
☒	☐ Clinical data
☒	☐ Dual use research of concern

Methods

n/a	Involved in the study
☒	☐ ChIP-seq
☒	☐ Flow cytometry
☒	☐ MRI-based neuroimaging

Eukaryotic cell lines

Policy information about cell lines

Cell line source(s)	Saccharomyces cerevisiae S288C derivatives
Authentication	Ploidy stability and auxotrophies
Mycoplasma contamination	NA
Commonly misidentified lines (See ICLAC register)	NA
